# Understanding the Social Determinants of Mental Health of Undergraduate Students in Bangladesh: Interview Study

**DOI:** 10.2196/27114

**Published:** 2021-11-02

**Authors:** Ananya Bhattacharjee, S M Taiabul Haque, Md Abdul Hady, S M Raihanul Alam, Mashfiqui Rabbi, Muhammad Ashad Kabir, Syed Ishtiaque Ahmed

**Affiliations:** 1 Department of Computer Science University of Toronto Toronto, ON Canada; 2 School of Computer Science and Mathematics University of Central Missouri Warrensburg, MO United States; 3 Department of Computer Science and Engineering Eastern University Dhaka Bangladesh; 4 Department of Computer Science and Engineering Bangladesh University of Engineering and Technology Dhaka Bangladesh; 5 Department of Statistics Harvard University Cambridge, MA United States; 6 School of Computing, Mathematics and Engineering Charles Sturt University New South Wales Australia

**Keywords:** Bangladesh, global south, social determinant, students, undergraduate, religion, women, mobile phone

## Abstract

**Background:**

The undergraduate student population has been actively studied in digital mental health research. However, the existing literature primarily focuses on students from high-income nations, and undergraduates from limited-income nations remain understudied.

**Objective:**

This study aims to identify the broader social determinants of mental health among undergraduate students in Bangladesh, a limited-income nation in South Asia; study the manifestation of these determinants in their day-to-day lives; and explore the feasibility of self-monitoring tools in helping them identify the specific factors or relationships that affect their mental health.

**Methods:**

We conducted a 21-day study with 38 undergraduate students from 7 universities in Bangladesh. We conducted 2 semistructured interviews: one prestudy and one poststudy. During the 21-day study, participants used an Android app to self-report and self-monitor their mood after each phone conversation. The app prompted participants to report their mood after each phone conversation and provided graphs and charts so that the participants could independently review their mood and conversation patterns.

**Results:**

Our results show that academics, family, job and economic condition, romantic relationship, and religion are the major social determinants of mental health among undergraduate students in Bangladesh. Our app helped the participants pinpoint the specific issues related to these factors, as the participants could review the pattern of their moods and emotions from past conversation history. Although our app does not provide any explicit recommendation, the participants took certain steps on their own to improve their mental health (eg, reduced the frequency of communication with certain persons).

**Conclusions:**

Although some of the factors (eg, academics) were reported in previous studies conducted in the Global North, this paper sheds light on some new issues (eg, extended family problems and religion) that are specific to the context of the Global South. Overall, the findings from this study would provide better insights for researchers to design better solutions to help the younger population from this part of the world.

## Introduction

Similar to many low-and middle-income countries [1,2], mental health among youth in Bangladesh is an overlooked and stigmatized topic [3]. More than 30% of adults in urban areas struggle with mental health–related issues [4]. At the same time, mental health facilities and services in Bangladesh are also insufficient [5]. The National Institute of Mental Health and Pabna Mental Hospital are the only major institutes that provide mental health treatment, constituting only 700 beds in total [4]. There are some other mental health institutes in or around the big cities as well; however, two-thirds of the total population living in rural areas have difficulty accessing mental health because of the lack of facilities in rural areas [4]. Support for nonserious mental illness is even poorer; the number of mental and emotional health support helplines is extremely low [6]. In addition, the general population still holds a traditional negative attitude and stigma toward those with mental health issues [7,8].

Proposing a path forward for addressing mental health challenges in the Global South is still at a rudimentary stage of development [9]. The social determinants of mental health [10]—social and cultural factors that deeply impact one’s mental health—have remained understudied in the context of Bangladesh. These factors vary depending on an individual’s age, financial condition, or social surroundings. However, in this paper, we focus particularly on undergraduate students, who constitute a nontrivial part of the population and are susceptible to mental health–related problems [11]. As various statistics and news reports corroborate the deep-rooted existence of suicide [12], depression [13], substance use [14], and extremism [15] among the young population in Bangladesh, and previous research indicates that such behavioral aberrations are often fueled by broader social, cultural, and political contexts in which one grows up and lives in [16-21], it is timely to investigate these underlying factors.

Concerns over the mental health of university students have been expressed in the Global North for some time now [22], although the same cannot be said about the Global South. According to a study conducted in a European country, around one-third of the first-year university students have been found to have mental health–related problems [23]. Several studies [23-25] have associated academic performance with mental health; that is, poor academic performance worsens mental health and vice versa. Other contributing factors for mental health deterioration include economic problems, high parental expectations, strained relationships, and poor lifestyle [8,26-29]. Several mental disorders are frequently observed among people aged between 14 and 24 years [30], many of whom are also university students. These students tend to encounter new experiences such as moving away from home and making adult financial decisions. In addition, many of them experience changes in their health behaviors [31]. Adapting to these changes sometimes becomes difficult, as students are also expected to perform their coursework and participate in exams. Failure to cope with any of these challenges may cause mental health difficulties [32].

These factors have already been identified as the social determinants of the mental health of students [10,31], although they are often specific to the context of the Global North. However, the different structures of society and culture in the Global South [33] may contribute to people’s mental health in other ways that are specific to the context of the Global South and have not been reported in previous studies. For example, in countries such as Bangladesh, extended families are far more prevalent where people live with their relatives (ie, uncles, aunts, cousins, or in-laws) [33]. Even when people are living in a nuclear family, social communication among relatives or neighbors is more frequent and cordial. As a result, maintaining regular communication with even distant relatives is viewed as an important social duty [34]. In addition, religion is a major component of people’s lives [34]. The concepts of religion and religious duties differ significantly from those in the Western context. A large portion of the population finds happiness through practicing religious duties, as religion offers their lives a sense of value and purpose. Religious identity dictates an individual’s social group and lifestyle choices [35]. The effects of complicated social relationships and disparate viewpoints on religion are yet to be understood in the context of students’ mental health in the Global South.

Through this work, we intend to explore the social determinants of mental health, particularly in the context of Bangladesh. In a country like Bangladesh, where social and family structures are hierarchical, traditional, and community based [10,31,36], social and family relationships play an important role in people’s daily lives, and recording one’s mental condition after interactions with family members and social peers seems to be a useful way of monitoring and improving their mental health. Past research suggests that patterns of mobile phone calls can reveal important information about an individual’s mental state and relationships with their friends and family [37]. In addition, mobile phone communication history helps one identify various forms of social closeness [38]. In recent years, Bangladesh has observed tremendous growth in mobile phone subscriptions. The total number of mobile phone subscribers in Bangladesh is approximately 160 million [39], which is almost equal to the total population of the country [40]. This shows that mobile phones are culturally relevant in Bangladesh. We see this as a research opportunity, as we can leverage the widespread use of mobile phones across the country to identify the social determinants of mental health of undergraduate students in Bangladesh.

One potential way toward forwarding the research on identifying the factors that affect mental health can be the use of reflective tools [41-43] that support self-monitoring of mood and emotions [44]. These mobile apps are suitable for measuring subtle emotions and capturing patterns of behavior in the long term [44]. A study conducted by Kauer et al [30] on youth aged between 14 and 24 years reported that self-monitoring one’s mood results in an increase of emotional self-awareness, eventually reducing depression. Another similar study [45], which helped participants identify the cause of their drinking habits (eg, relation with romantic partners), showed success in tracking information about alcohol consumption and its effect on mental behavior. Kiekens et al [25] explored the impact of mood-tracking apps in clinical settings, and their pilot study received positive feedback from both participants and therapists, as participants could review their relationships with their family and peers. Pollak et al [46] suggested that reflective tools with the option to provide self-reported emotions can give good insights into human behavior. In the psychology literature, ecological momentary assessment [47] supports the existence of a theoretical framework that demonstrates the importance of such in-the-moment mood assessment. The longitudinal nature of data in these tools gives users an opportunity to examine the effects of different life situations on their mental state, as users can document their moods and emotions at various times and do independent research on their historical data later [47]. Pollak et al [46] claimed that providing information about one’s emotion and mental state at random times of the day can lead to improvement in social behavior. Other studies [48-52] have also shown the promise of self-reported mood-tracking apps in mental health research.

In this work, we designed, developed, and deployed a mood-tracking Android app that could be used as a reflective tool to record and reflect on past interactions with social peers. We conducted 2 semistructured interviews with our participants—one before and one another after using the app to understand the impact of our app on their mental health. Our primary goal is to progress the research toward identifying the factors that affect the mental health of Bangladeshi undergraduate students. However, we do not make any causal claims about these factors; instead, we present the findings from our interviews that give insights into how certain factors manifest in the specific context of Bangladesh, as well as shed light on some new issues that were rarely discussed before. As a secondary goal of the study, we want to observe the effect of the self-monitoring tool and see whether our participants take any action to improve their mental health, despite no explicit recommendation provided by the app.

## Methods

### Participants

The study was conducted on 38 participants aged between 19 and 27 years. At the time of the study, all the participants were full-time undergraduate students. Participants were recruited through snowball sampling [53] via social acquaintances, classroom announcements, and word of mouth. Students were recruited from 7 universities in 2 major cities in Bangladesh: Bangladesh University of Engineering and Technology (BUET), Eastern University, Thengamara Mohila Sabuj Sangha Medical College, Dhaka College, Rajshahi University, City College, and Bangladesh University of Business and Technology. 

Our participants included 30 traditional and 8 nontraditional students (P1, P5, P24, P25, P26, P30, and P35). In Bangladesh, traditional students join university immediately after receiving their high school diploma, whereas most nontraditional students attend university after receiving 2-3 years of technical vocational training (eg, application of different computer software in official contexts) and a few years of job experience [54]. Table 1 provides information about the participants’ gender, age, and institution.

**Table 1:** Information about the participants^a^.

Participant	Gender	Age (years)	Institution	Student type
P1	Male	25	EU^b^	Nontraditional
P2	Male	24	EU	Traditional
P3	Male	22	EU	Traditional
P4	Female	24	EU	Traditional
P5	Male	25	EU	Nontraditional
P6	Female	24	EU	Traditional
P7	Female	25	EU	Traditional
P8	Male	23	BUET^c^	Traditional
P9	Male	23	BUET	Traditional
P10	Male	24	BUET	Traditional
P11	Male	23	BUET	Traditional
P12	Male	23	BUET	Traditional
P13	Male	23	BUET	Traditional
P14	Male	24	BUET	Traditional
P15	Female	23	BUET	Traditional
P16	Female	23	BUET	Traditional
P17	Female	23	BUET	Traditional
P18	Female	20	BUET	Traditional
P19	Male	23	BUET	Traditional
P20	Female	22	RU^d^	Traditional
P21	Male	22	DC^e^	Traditional
P22	Male	21	DC	Traditional
P23	Male	22	DC	Traditional
P24	Female	24	EU	Nontraditional
P25	Male	27	EU	Nontraditional
P26	Male	20	EU	Nontraditional
P27	Female	22	CC^f^	Traditional
P28	Male	19	RU	Traditional
P29	Male	20	RU	Traditional
P30	Male	25	BUBT^g^	Nontraditional
P31	Female	19	TMC^h^	Traditional
P32	Female	19	TMC	Traditional
P33	Female	19	TMC	Traditional
P34	Female	19	TMC	Traditional
P35	Male	26	EU	Nontraditional
P36	Female	23	EU	Traditional
P37	Male	23	BUET	Traditional
P38	Female	20	TMC	Traditional

^a^P36-P38 did not take part in exit interviews.

^b^EU: Eastern University.

^c^BUET: Bangladesh University of Engineering and Technology.

^d^RU: Rajshahi University.

^e^DC: Dhaka College.

^f^CC: City College.

^g^BUBT: Bangladesh University of Business and Technology.

^h^TMC: Thengamara Mohila Sabuj Sangha Medical College.

### Study Design

#### Overview

We designed and developed *HWC* (How Was the Call?)—a mobile phone app that is a self-reporting tool to record and reflect on past phone calls. We conducted 2 semistructured interviews with each participant, one before (baseline interview) and another after (exit interview) using the app. Between these 2 interviews, the participants were asked to install and use the app for 3 weeks. The study was approved by the institutional review board of the authors’ institution.

#### Baseline Interview

During this interview, we asked the participants about their general mental health and the reasons behind their mental stress, depression, or frustration; the frequency of experiencing mental health issues; the relationships that cause them; and their coping strategies. As English is the primary medium of instruction in their institutions, all of our participants were proficient in English. However, to collect spontaneous responses and have an in-depth discussion, we conducted the interviews in their native language Bengali, the official language of Bangladesh [55]. We occasionally used English words for different mobile phone and mental health–related terms.

Interviews were conducted both in person and through the Zoom videoconferencing platform. All interviews were audio recorded with the consent of the interviewees. Interviewers ensured appropriate levels of empathy and rapport during the interviews. Time-outs were offered if the participants asked for them.

The average length of the interviews was 25 minutes (SD 13.76; SE of the mean 3.97).

#### The 3-Week Study With the HWC App

**Figure 1:**
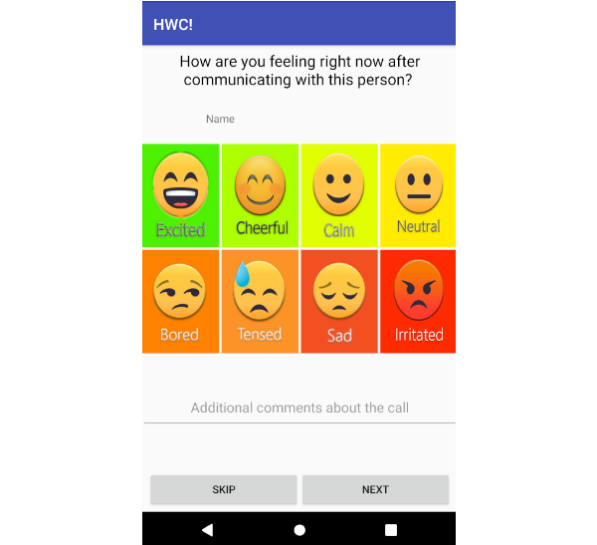
Interface for providing feedback after each call. HWC: How Was the Call?

After the baseline interview, participants were requested to use the HWC app for 3 weeks. Study personnel helped the participants install the app on their smartphones and instructed them on how to use it. We designed the app to run in the background and pop up a survey at the end of each phone call. The survey asked the participants to describe their mood by choosing from 8 emotions (Figure 1): *excited, cheerful, calm, neutral, bored, sad, tense,* and *irritated*. These 8 emotions cover the circular space of the valence-arousal dimension [56], a common framework for recording emotional experience, which assumes that all human emotions are distributed in a 2D space. Previous works [37,44,46] show that this approach of asking emotions from the valence-arousal dimension is particularly suitable for apps where users need to give frequent quick inputs on their mood, and it offers a reliable tool for researchers to analyze user behavior. One might argue that self-reported moods might not cover all mood archetypes; however, a vast amount of psychology literature [37,44,46] validates the use of our or similar to our self-report methods. In addition to the ease and convenience of the implementation of these methods, self-reported methods provide users with enough control over their interaction with the system, which does not make them feel they are part of an experiment and instead generate data closer to the real world [44,57]. These approaches inspire spontaneous responses from people and are helpful for capturing fluctuations in behavior and symptoms [57].

In addition to the 8 emotions, we also provided the participants with a textbox to describe their feelings in more detail. Although we could have asked for more inputs from users (eg, how strong is the emotion?), our app asked for user input every time they made or received a call. Hence, asking for too many inputs from users after each phone call would require a high cognitive burden that might alter emotion and reduce long-term engagement [44]. However, a participant can choose to skip and provide no information. This app does not require any internet connection and stores all information in the local memory of the mobile phone. As a result, there is no risk of participants’ information going public.

**Figure 2:**
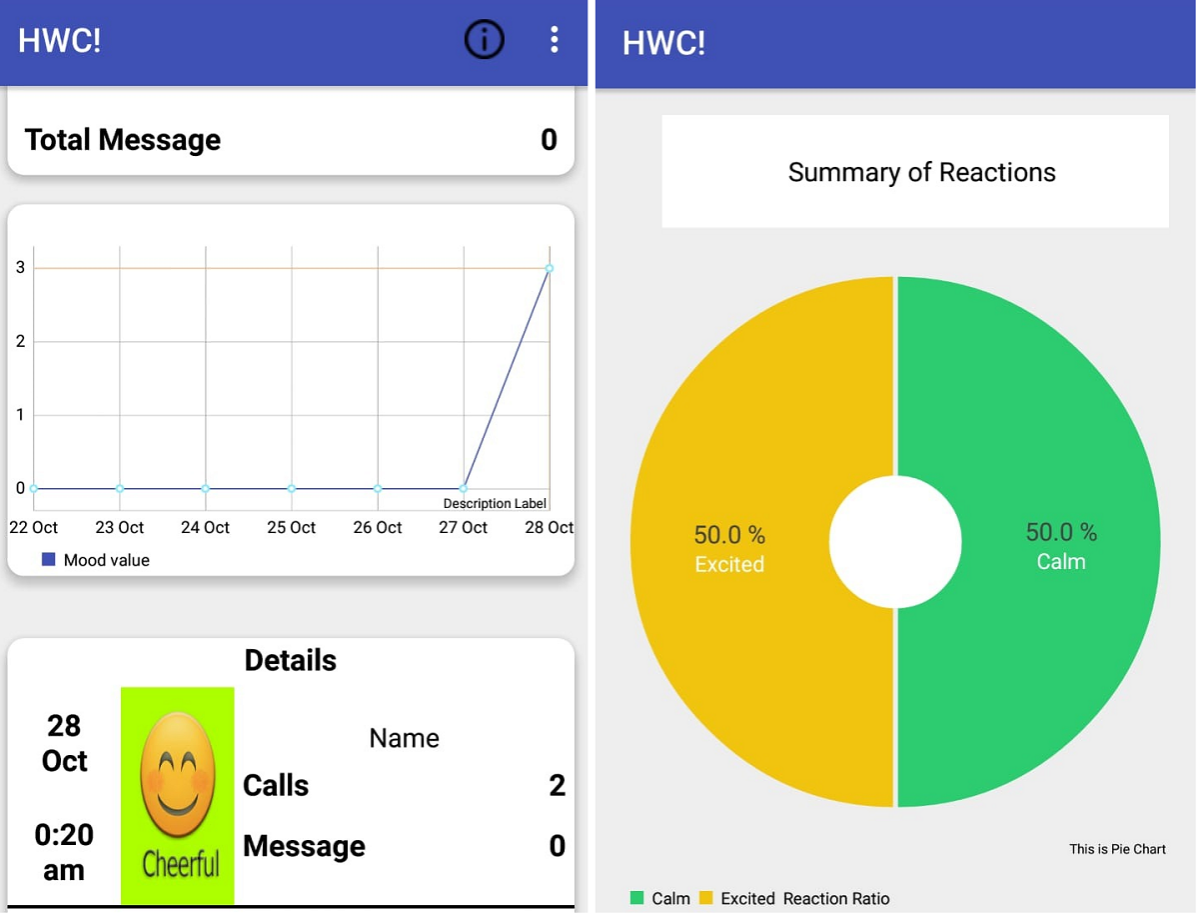
Interfaces for analyzing call history and mood. HWC: How Was the Call?

Once the participants described their feelings, they had the option to view their data later. The participants could review their call history and see how they felt after each call (Figure 2). They could also view a visual summary of their reactions over a period of time with the help of a pie chart (Figure 2). Although the app let participants see their information, it did not provide any sort of feedback or recommendation to alter the mood.

The flow of the app is as follows:

1. The participant installs the app.
2. Every time the participant makes or receives a regular phone call, the app starts its operation.
3. After the call ends, the participant is prompted to provide feedback and choose from 8 options (Figure 1). A textbox is provided for additional written comments. A participant can take any of the following 2 steps:
  After providing the information, the participant can press Next. This stores the information in the local memory of the mobile phone, and the app terminates.
  The participant can choose not to provide any information by pressing Skip. The app then terminates.
4. The participant checks the call history and reviews their emotional status after each conversation at any time (Figure 2).

Participants who were worried about their data being stored outside the phone (public server and database) or shared with a third party were ensured that their call history and mood descriptions would only be stored in their mobile phone memory, and they were even allowed to see the source code of the app. In fact, 2 participants who were familiar with Android app development requested to review our source code, and we allowed them to do so. We did not collect specific information about the number of calls received by each participant for privacy reasons.

#### Exit Interviews

Exit interviews were arranged after the participants had used the HWC app for 3 weeks. In these interviews, participants described their experience of using the app to track their moods. The questions were more specific in this session, where we asked them to highlight the major incidents that they had recorded over the past 3 weeks. We also asked them how the app had shaped their opinions and attitudes toward other people in their social circle. The setting of this interview was the same as that of the baseline interview. The average length of the interviews was 17 (SD 7.43, SE 3.03) minutes. Despite our best efforts, we could not reach 3 participants (P36-P38) in this session because of their unavailability, resulting in a data set of 35 (male: 21/35, 60%; female: 14/35, 40%) interviewees.

### Data Analysis

We first transcribed the audio recordings from both sets of interviews and then translated them into English. Next, we performed a thematic analysis of our data based on the Boyatzis framework for code development [58]. From our analysis of the baseline interviews, we tried to explore the major social determinants of mental health among undergraduate students in Bangladesh. The responses of the participants during the exit interviews demonstrated the effectiveness of the app in monitoring their mental health and its impact on identifying the specific personal issues they had been dealing with. The results of the analysis are described in the next section.

## Results

### Overview

In this section, we first describe the findings of our baseline interviews by highlighting the factors that affect the mental health of our participants. Then, we report the impact of our app on helping participants monitor their mental health. Finally, we describe the measures that were taken by our participants to improve their mental health, although no recommendation was provided by the app.

### Factors Affecting Mental Health

#### Overview

Through our interviews, we identified 5 factors that affected our participants’ mental health the most. Table 2 illustrates the distribution of these factors based on how many participants reported them. Several participants reported multiple factors. Each of these factors is presented in detail later.

**Table 2:** Distribution of the factors that were reported by the study participants (n=35).

Factor	Participants, n (%)
Academic performance	27 (77)
Family	19 (54)
Job and economic condition	17 (49)
Religion	11 (31)
Romantic relationships	9 (26)

#### Academics

Academic standing was the most discussed theme during the interviews. Of the 35 participants, 27 (77%) noted that academic performance had a major impact on their mental health. Their main concern was the stress and depression created by assignments, class tests, projects, and final exams. One participant said:

I feel depressed on the evening before my exams. I get very tensed and start to think too much about the upcoming exam. Often I feel uncertain whether I will be able to answer all the questions or not. Sometimes I am too confused and start to panic about my preparation. I call my classmates and ask them about their preparation. Although they tell me that they have not prepared very well either, I assume otherwise and get more depressed.

P4

Another participant (P11) further added that he had been stressed because of his final-year academic thesis. Even during vacation, he had to work on his thesis. Other participants were more concerned about their academic grades. They thought that their results were not satisfactory, and they might struggle in the job market. As mentioned by another participant:

I get frustrated when I try to read in the evening. I think about my future career and feel that I am not doing enough. I have a poor CGPA, will anyone give me a job? At times, it seems that I am a complete failure, which makes me frustrated.

P2

A similar sentiment was reflected in the response of P10. In his opinion, despite putting in his best efforts, he had not been able to achieve his desired grades, which resulted in mental depression and frustration. He said the following:

I have been getting poor grades in exams in consecutive semesters. Despite trying my best, I am not being able to improve my grades. It’s not that I’m not trying, but nothing seems to work.

P10

Another participant (P17) expressed her concerns over academic deadlines. She felt that procrastination contributed to a decline in her mental health. She tended to start working at the 11th hour to complete her assignments; however, before that, she could not focus on anything else either, as she constantly kept reminding herself about the deadline. She felt that this sort of behavior put her in *some kind of loop*, which was the main reason behind her stress. P25 also shared similar frustrations; however, his reason was that he did not have time to focus on his studies because of his full-time job. He also felt that he was in a loop as he joined the program to have a BSc degree and have promotions in his current career; however, at the same time, his career prevented him from concentrating on his studies.

#### Family

Of the 35 participants, 19 (54%) mentioned family as a reason for the decline in their mental health. However, the underlying reasons varied significantly. As the participants were from different backgrounds in terms of social and economic status, their problems were also different in nature. For example, one participant (P1) said that he had lost his parents at the age of 15 years. As the eldest child, he had to pick up many responsibilities since then. Although he was quite happy to perform his responsibilities, he constantly felt that he had not been doing enough for a better upbringing of his younger brother. He said the following:

When my mother died, my younger brother was still a baby. We lost our father after a few years, so his only guardian now is me. I am always tense about his education. Sometimes my brother does not listen to what I say. Those times are very hard for me. Whenever he fails at anything, I feel genuinely frustrated.

P1

The participants who were married (P5, P6, P7, and P24) talked about their spouses and in-laws when discussing mental issues. One male student (P5) with a full-time job said that he was always concerned about financial stability, as his wife did not have a job. Owing to his busy life, he missed many family events, and although the reasons were genuine, his in-laws felt that he did not give them enough attention. Of the 35 participants, the 3 (9%) recently married female participants (P6, P7, and P24) discussed at length about their relationships with in-laws and how this affected their mental health. In Bangladesh, it is a common practice for women to move to their husbands’ houses after the wedding. As extended families are still a cultural norm in Bangladesh [33], a new wife needs to build a relationship with her husband’s family. These issues were pointed out by both P6 and P7. P7 said the following:

I am currently having issues with my new family. It is not any particular person, but their overall attitude. I have to be careful about my every action because if they don’t like anything, they directly call my parents to complain about me. What hurts me more is that my own parents do not support me in this case. The relationship dynamic with my in-laws is perhaps the biggest cause of stress in my life currently.

P7

Some other participants mentioned that as they were too busy with their academic life, they got stressed whenever they were burdened with any additional family responsibilities, including sending money and taking care of sick family members. Approximately 6% (2/35) of participants (P13 and P27) said that simply talking about family problems through the mobile phone was stressful for them.

#### Job and Economic Condition

Of the 35 participants, 5 (14%) participants (P1, P5, P25, P30, and P35—all nontraditional students) had full-time jobs during the study period, and all remarked that work-related issues affected their mental health. P5 talked about the competitive environment of his office—how everyone there was under huge pressure to perform and complete the tasks assigned to them. He said the following:

A full-time employee has a lot of responsibilities. You have pressure from your boss – pressure of meeting deadlines, pressure of performing better than your colleagues. Even at midnight, I receive calls from my boss. This makes me think that life without mobile phones would have been much better. At least your boss could not have reached you after office hours. [...] When I fail to meet a deadline, I get anxious. You fail, which means that you will have a lesser chance of being promoted and there are many other people waiting to grab that opportunity. I used to think competition exists only in colleges, but it is certainly more in professional life.

P5

Some of our participants mentioned that their families were solvent, and they did not have an urgency to get internships or jobs. However, they still felt the pressure to look for a job, as their peers had been doing the same. One participant (P14) highlighted this issue as a major reason for the decline in his mental health. Some participants, on the other hand, got confused about their career choices. In Bangladesh, career counseling is not recognized as an important activity at universities [59,60]; thus, many students face a dilemma regarding their career choices. This is reflected in the response of a business major:

I think about my future all the time. I am finding it really difficult to choose between two specific options: doing an MBA or preparing for job interviews. I try to consult my seniors, but even they do not have a clear career path for this program.

P6

As mentioned before, of the 35 participants, 12 (34%) were from BUET, which is typically regarded as the most sought-after academic institution in the country. As a result, they had a high demand as instructors in local coaching centers [61] or as private tutors. In fact, all of our participants from BUET, except P9, had a part-time income source as private tutors. We found a few tutoring-related issues that affected their mental health. For example, P10 mentioned that he received many calls from his students during his own study hours, and concentrating too much on his students might be a reason for the deterioration of his mental health as it affected his own grades. P11 also expressed similar concerns; however, for him, making money was a bigger priority than obtaining good grades, even as a student. In his opinion, he became frustrated when he did not have a good number of students to be able to send enough money to his family. In fact, he was mentally quite happy during our study period as he had been earning more money than ever.

#### Religion

Of the 35 participants, 11 (31%; all Muslims) discussed the role of religion in their mental health. One of them (P1) said that when he missed the Fajr prayer (the first of the 5 daily prayers performed by a practicing Muslim) at dawn for oversleeping, he did not feel well for the rest of the day. However, he eventually calmed himself by thinking that an unwillingly committed negligence would not be punished and a good act later in the day would compensate for the earlier remission. He further added the following:

I believe in Tawhid [the concept of monotheism in Islam], so I believe the whole universe has been created by our God. He has given us certain duties to perform, and sometimes when I fail to do any of those, I get a little bit stressed out. [...] I have another issue pertinent to religion. In a society, different types of religions coexist and I have certain expectations from the believers of other religions. At times, those expectations are not met and I face trouble in interacting with people from other religions, which bothers me a lot mentally.

P1

P27, who also expressed similar sentiments, felt that each problem in life could not be shared with family. That is why he had turned to God with Whom he feels that every problem can be shared and discussed. However, all of these participants highlighted that they performed Salah (prayer) to seek happiness. In addition, P4 and P24 said that they regularly read the Quran (the main religious text of Islam) to relieve mental stress.

#### Romantic Relationships

Of the 35 participants, 9 (26%) talked about their romantic relationships when discussing mental health. The 3 married participants, including P7, who was not happy with her in-laws, appreciated the support they received from their spouse in conjugal life. However, all of them confessed that they needed to think about their actions thoroughly, as those might affect their spouse. This constant pressure of remaining careful and alert creates a stressful family environment for them.

The unmarried participants (P9, P11, P13, P21, P28, and P31) described their past and present romantic relationships. One participant correlated his stress and frustration with his relationship with his partner:

I think my major source of stress and depression is my relationship with my girlfriend. We have been in the relationship for a while now, and I always try to make her happy in any way I can. Even then, I sometimes notice that the value of my opinion matters less very little in our relationship. Additionally, I have a tendency to compare my financial status with my girlfriend’s, which also makes me sad or in some cases, jealous.

P21

Another participant (P9) pointed out a different aspect of relationships, saying that his ex-girlfriend had issues of emotional dependency and that he had to spend a significant amount of time throughout the day talking with her over the phone. He felt relieved to get out of the relationship as he was unable to cope with the habit of spending too much time on the phone. P11, who had recently broken up with his girlfriend, was visibly distressed because of the failed relationship. However, one participant (P13) attributed the supportive nature of his girlfriend to his sound mental health, saying that he always found his partner by his side when he was stressed.

### Monitoring Mental Health

Having described the factors affecting the mental health of our participants, we now highlight the effectiveness of our app in monitoring their mental health. Through our app, participants were able to track their own behavioral patterns by regularly recording their information. All of our participants agreed that the moods shown in the app represented the entire spectrum of their real-life emotions and made them more conscious about their mental health:

Each time I finish a call, the app asks me how I am feeling. This makes me think about my mental health for a few moments, which I would not have done previously. The app makes me more aware of my mental health.

P1

Another participant (P2) liked the feature of reviewing the mood data. He reported that during the course of 3 weeks, he had periodically analyzed his mood report, and in doing so, he could identify some patterns. For example, he had realized that he felt more tense at night.

Our participants also mentioned that the app helped them in identifying the positive and negative influences in their lives. As a result of using the app, they were able to more consciously identify the positive influencers—persons with whom they are close in real life but never previously thought of as positive catalysts for the betterment of their mental health. For example, P5 acknowledged this revelation in the following way:

Through this app, I could identify those people who make me happy and I realized that even during face-to-face conversations, this same group of people makes me feel more comfortable. I could make this explicit connection by using this app.

P5

Similarly, some participants noted that the use of the app was helpful in identifying persons who caused mental stress in their lives. Of the 35 participants, 2 (6%) participants (P6 and P26) mentioned that the emojis were quite helpful in this regard as they could properly categorize their mental state by using those emojis. They were able to identify the conversations that had a negative impact by looking at the emojis for *sad* and *irritated* reactions. A couple of other participants (P2 and P4) also explicitly recognized the importance of the emojis. Choosing emojis after each phone call gave P4 a sense of excitement similar to *playing games on a mobile phone*, which she viewed positively.

Some of our participants reported how the app assisted them in precisely identifying the reason for their mental stress. For example, during the baseline interview, P11 said that the final-year thesis had been his primary academic concern. By using the app, he was able to understand that his supervisor had been pushing him a lot for the thesis. After each phone call with his supervisor, he was either *tense* or *sad*. At times, he felt *irritated* after receiving several calls regarding the improvement of the thesis document. Similarly, before using the app, P13 already knew that talking with his family members over the phone had been stressful for him. After using the app for 3 weeks, he realized that things got worse when economic problems were discussed during any conversation but at other times, the conversations were not as stressful. Of the 35 participants, 7 (20%) participants (P3, P10, P18, P22, P28, P30, and P35) specifically mentioned that the app helped them pinpoint the exact cause of their mental stress.

One participant (P12) provided important feedback regarding the textbox feature for writing additional comments. He thought that the overall mood was not entirely dependent on the conversation, as it got affected by other factors (eg, weather or an upcoming exam). He particularly liked the option of having a textbox to be able to record the potential reasons behind certain emotions. In his opinion, the written comments were helpful to better understand the contexts when he reviewed them later.

### Impact on Mental Health Improvement

As the use of the app enabled our participants to better identify the persons or events that had a negative impact on their mental health, they could also take some measures for improvement. About two-thirds (24/35, 69%) of our participants said that they had tried to change the frequency of contacting those people who were having a negative effect on their mental health:

The app helped me identify the people who were causing stress. As I checked the call patterns, I tried to understand why I was not feeling comfortable talking with them. What I did was – I tried to improve my relationship with them. Some attempts succeeded, while others did not. I have started avoiding those persons with whom I could not develop a better relationship.

P6

Responses from P14 also revealed similar adjustments. By using the app, he realized that he had been in touch with some friends who were detrimental to his mental health as phone conversations with them were making him *sad*. He reduced the frequency of phone conversations with those people and reported that he had already started feeling better. P29 was also able to identify such people in his life. Apart from having fewer phone conversations with them, he started changing his tone and approach in physical meetings:

After I found out who those friends were, whenever I met them I explicitly tried to show that I am not interested in talking with them. I deliberately made the conversations shorter and removed the friendly approach from my tone.

P29

However, our participants also mentioned that it is impossible to avoid certain persons in academic or professional lives (eg, supervisors and managers), and they had to pick up phone calls from those persons or call them for important academic or professional reasons. At times, they also had to pick up specific calls for courtesy and modesty. Some participants mentioned that the app had been helpful in these cases as based on previous mood patterns recorded through the app; they were mentally prepared for certain conversations to have a potential negative impact on their mental condition:

In my office, I often need to contact many colleagues and those conversations can’t be avoided. But now the app has let me know that after some of those conversations, I’ll not feel cheerful. As I know this beforehand, the impact is less severe.

P5

One participant (P1) said that the app helped him control his temper. He talked about a particular relative who used to call at inappropriate times for mundane conversations. As P1 felt *annoyed* after receiving that relative’s call, he tried his best to remain calm when talking with him. P1 also mentioned that the app made him aware of his attitude toward a few other family members as well. Another participant (P10) said that the app offered him the opportunity to find a new support system within his own family:

I have a large family with many sisters. As I am under huge academic pressure, I cannot contact them as frequently as I would like to. But after using the app, I found that my stress relieves a lot when I share my problems with my sisters. They give wonderful advice, too. I have decided to communicate more frequently with them.

P10

The app also seemed to have a positive impact on the relationship status of a participant (P13). Although P13 had a stable and healthy relationship and used to share his problems with his girlfriend even before using the app, he noted that the app reinforced the same notion about the relationship, as he often felt *cheerful* or *calm* after talking with her.

## Discussion

### Principal Findings

#### Social Determinants of Mental Health of Undergraduate Students in Bangladesh

Our baseline interviews identified several factors that affect the mental health of students in Bangladesh. Our findings demonstrate some differences between Western countries and Global South countries such as Bangladesh with regard to social determinants of mental health of the young, college-going population. Although some of the factors (eg, academic performance [23-25,62] and economic conditions [28,63]) we identified in our study have already been pointed out by previous studies conducted in Western contexts, issues such as extended family (in-laws) problems and religion have not been reported before.

Female students who go for higher education need to overcome the barriers created by the local society [64,65]. This situation is even more difficult for married students. In a country where >52% of children get married before reaching 18 years of age [66,67], it is expected that a large number of undergraduate students, especially females, would be married. We had 3 married students in our study, 2 of whom were female. According to the local culture of Bangladesh, the bride moves in with the groom’s family after marriage. New brides often need to adjust to a different family and adapt to their culture and rules. When discussing mental health, both of our female married participants talked about their struggle to adapt themselves to their husbands’ families. This struggle to adapt with in-laws or extended families should be investigated in the context of students from other countries in nearby regions (eg, India and Pakistan), as underage marriage and discrimination against women likely exist in those countries as well [2,68].

Religion has been an integral part of the social, political, and economic environments of Bangladesh [34]. The state religion of the country is Islam [69], and 90.3% of the total population identifies themselves as Muslim [40]. Religious identity is a significant factor behind happiness in Bangladesh [34]. This view aligns with one of our participants’ stress behind the failure to perform religious duties. Some other participants also briefly mentioned that they performed regular prayers to seek mental happiness.

Although economic conditions have been a factor discussed in previous studies, the context of Bangladesh is somewhat different. In the local society, a male is expected to support his parents and, in some cases, the extended family. Therefore, students at the undergraduate level, especially male students, start worrying about their financial status. Families also start sharing financial problems with them, indicating that the students now need to contribute to their families. For example, P11 and P13 felt stressed and frustrated when they failed to send enough money to their homes. However, we did not get such reports of stress over the family’s economic status from any of our female participants.

Apart from traditional students, we also had 23% (8/35) nontraditional students (female: 1/8, 13%; male: 7/8, 87%) in our study. Like regular students, they mentioned issues about coursework and exams, but all of them pointed out other issues as well. Of the 8 nontraditional students, 4 (50%) were married; therefore, these participants mentioned their struggle to measure up to their in-laws. Even the male married student (P5) talked about how his inability to attend family functions made his in-laws unhappy. Of the 35 participants, 5 (14%) male participants (P1, P5, P25, P30, and P35) explained their job life in detail and expressed that they found it very difficult to manage a full-time job with academic life. Their conversations (particularly that of P25) hinted that they were expected to do a full-time job as they were getting old; however, at the same time, they had to be serious about their academic coursework, as not having a bachelor’s degree hindered their progress in job life.

#### How the App Helps Identify One’s Own Social Determinants

The most commonly appreciated theme regarding our app was its ability to help users observe their mental states. In the baseline interviews, many of our participants had rough ideas about the main factors affecting their mental health; however, using the app for 3 weeks helped them pinpoint the exact reason. During this period, our participants constantly put their emotions after each call. As our app provided visual analysis of call history through graphs and pie charts, they could interpret those in their own way. Our participants were able to notice which calls evoked negative sentiments and acted accordingly. For example, the app helped several participants identify that academic supervisors or some specific family members were the reason for their stress. Conversely, the app also helped them identify the relationships that had a positive impact on their mental health. One participant further discovered the temporal aspect of mental health by using the app.

Similar to other reflective tools [41-43] with the option of providing self-reported emotions, a benefit of our app was that the participants could analyze their calls and moods in any way they wanted. Looking at the graphs and charts, they could review their moods over multiple days, or they had the option to analyze their conversations with a single individual. Although the participants mostly talked about identifying positive or negative relationships in our study, we also had people who identified other patterns of their mental states (eg, P2 felt more stressed at night). P13 identified that talking with family only stressed him when the conversation was about financial problems. Overall, the flexibility that we provided to our participants regarding the interpretation of their calls and emotions enabled us to have a diverse set of insights into their mental state.

Although the app was used only by Bangladeshi students in our study, apps such as these can be used in a universal context. In our study, students could identify the factors causing a negative impact on their mental health. These factors may be different in the context of people from other cultures; however, everyone can realize their own factors by using the app for a prolonged period. Even in our study, the participants reported a range of factors using the same app. We believe that this app can be considered as a variant of a digital diary [70], which can act as a support system for mental health [11]. However, we note that the topic of using the textbox to describe the phone calls had come up only once in our study.

#### Actions Taken by Users

After identifying some of the potential factors, the participants in our study took some measures to improve their mental health. These measures were not suggested or recommended by our app; rather, they were taken by the participants on their own. For example, 24 students reported that they changed the frequency of contacting the people whom they found to have a negative impact on their lives. Among these participants, some tried adjusting their stressful relationships, whereas others started avoiding interactions altogether. In cases where avoiding a person was not a feasible option because of professional reasons (eg, colleagues or supervisors), the app helped the participants prepare themselves mentally for a potential negative conversation.

It should also be noted that the app cannot address all the cultural or social aspects of human life. For example, our mobile app cannot directly assist female students regarding the challenges they face in their day-to-day lives. Treating women as inferior or violence against them are deeply rooted social problems, and no mobile app can solve these issues immediately [65]. Although our app can provide some information about the underlying reasons in contexts such as this, it cannot go beyond to fix those problems.

### Limitations and Future Work

Our paper does not make any causal claims about the social determinants of mental health (eg, we do not make claims that marriage causes mental frustration to young women). We only describe the findings of our interviews, and the factors we reported may not be causally related to mental health. Other hidden, unmeasured variables may play a role. However, we believe our reported factors will pave the way for future work that will dive deeply into investigating the underlying mechanisms of mental health, particularly the ones that came up for the first time in this study. We believe that follow-up work can then inform the theories of the mechanism of mental health. These theories can then be verified with actual field experiments to quantitatively find causal relationships.

Although we included students from 7 universities that offer different standards of education, future work should interact with students from even more diverse backgrounds. Our participants predominantly lived in 2 major cities, and students from universities in semiurban or rural areas might face other factors that have not been identified in this study. It would also be interesting to observe how this different demographic group struggles with the factors reported in this study and whether their perceptions about using a mobile app to monitor mental health would be any different.

Finally, there is a growing tendency among students to use social media platforms, such as Facebook Messenger, WhatsApp, or Emo, for having longer conversations. As the internet has started reaching even the most remote parts of the country [71], data plans and broadband internet are not as costly as before, and calling over the internet is a much cheaper option in many cases. Our participants also mentioned that they used several platforms to contact friends or family members. Future studies can be designed on these internet platforms to monitor the mental health of the young, college-going population of Bangladesh in a more comprehensive way.

### Conclusions

In this work, we aimed to progress the research toward identifying the social determinants of mental health among undergraduate students in Bangladesh. We designed and deployed an Android app among our participants to help them record and later reflect on the mobile phone conversations with their friends, family members, and academic or professional correspondences. Our app assisted the participants in pinpointing the exact relationship or factor that had a detrimental effect on their mental health. Although some of these factors have been reported in previous studies conducted in the Western context, we identified several new factors, including religion and extended family affairs, which are pertinent to the society of Bangladesh. Although our app does not provide any recommendations from its side, some participants took independent measures to improve their mental health. However, in some cases (eg, extended family problems), despite identifying the problem, participants could not find an appropriate solution; however, they could better prepare themselves to cope with that specific issue. Taken together, our study provides useful perspectives on and insights into the mental health of undergraduate students in Bangladesh, and we hope our findings can help researchers design better solutions to improve the mental health of the younger population from this part of the world.

## Abbreviations

BUET: Bangladesh University of Engineering and Technology
HWC: How Was the Call?
